# Minimally Invasive Approach for Replacement of the Ascending Aorta towards the Proximal Aortic Arch

**DOI:** 10.3390/jcm13113274

**Published:** 2024-05-31

**Authors:** Florian Helms, Ezin Deniz, Heike Krüger, Alina Zubarevich, Jan Dieter Schmitto, Reza Poyanmehr, Martin Hinteregger, Andreas Martens, Alexander Weymann, Arjang Ruhparwar, Bastian Schmack, Aron-Frederik Popov

**Affiliations:** 1 Division for Cardiothoracic-, Transplantation- and Vascular Surgery, Hannover Medical School, Carl-Neuberg-Str. 1, 30625 Hannover, Germany; 2 Clinic for Cardiac Surgery, University Clinic Oldenburg, 26129 Oldenburg, Germany

**Keywords:** minimally invasive surgery, aortic surgery, ascending aorta replacement, proximal aortic arch replacement, hemiarch replacement

## Abstract

**Objectives:** In recent years, minimally invasive approaches have been used with increasing frequency, even for more complex aortic procedures. However, evidence on the practicability and safety of expanding minimally invasive techniques from isolated operations of the ascending aorta towards more complex operations such as the hemiarch replacement is still scarce to date. **Methods:** A total of 86 patients undergoing elective surgical replacement of the ascending aorta with (*n* = 40) or without (*n* = 46) concomitant proximal aortic arch replacement between 2009 and 2023 were analyzed in a retrospective single-center analysis. Groups were compared regarding operation times, intra- and postoperative complications and long-term survival. **Results:** Operation times and ventilation times were significantly longer in the hemiarch replacement group. Despite this, no statistically significant differences between the two groups were observed for the duration of the ICU and hospital stay and postoperative complication rates. At ten-year follow-up, overall survival was 82.6% after isolated ascending aorta replacement and 86.3% after hemiarch replacement (*p* = 0.441). **Conclusions:** Expanding the indication for minimally invasive aortic surgery towards the proximal aortic arch resulted in comparable postoperative complication rates, length of hospital stay and overall long-term survival compared to the well-established minimally invasive isolated supracommissural ascending aorta replacement.

## 1. Introduction

Over the last two decades, minimally invasive techniques have developed rapidly in cardiac surgery, and the feasibility and safety of minimally invasive cardiac surgery have been demonstrated for different cardiac procedures [1,2]. Likewise, minimally invasive approaches have gained immense importance in aortic surgery as well and have become the standard approach for limited operations of the aortic valve, aortic root and ascending aorta in many centers [3,4,5,6,7]. For this, numerous techniques have been developed including different types of upper hemisternotomies as well as sternal sparing thoracotomy approaches [8,9,10,11,12,13,14]. Currently used surgical techniques and outcomes for minimally invasive surgery of the ascending aorta have been described in detail in a recent review from our working group [3]. In matched analyses, comparable results regarding safety outcomes, mortality and hospital stay have been reported for minimally invasive aortic root and ascending aorta replacement compared to the standard full sternotomy techniques [8,9,12]. In a recent study, Angerer et al. reported longer operation times but no differences in hospital stay or long-term outcome in minimally invasive ascending aorta replacement compared to full sternotomy [15]. Following this development, the minimally invasive approach has been continuously expanded to address more complex aortic pathologies through limited access sites. As one of these techniques, surgical repair of more distal parts of the ascending aorta and proximal aortic arch via upper hemisternotomies is now performed with increasing frequency [16]. In the current literature, this technique is also referred to as ‘hemiarch replacement’ [17]. However, including replacement of the proximal aortic arch in the repair distinctively increases the complexity of the operation compared to isolated replacement of the ascending aorta alone. Since cross-clamping of the ascending aorta proximal to the brachiocephalic trunk is not feasible for proximal aortic arch replacement, hypothermic circulatory arrest is generally inevitable and selective cerebral perfusion has to be established through minimally invasive access, which can be challenging depending on the cannulation strategy and access technique [3,9,10].

Consequently, while the results of the now well-established minimally invasive procedure to replace the aortic root and proximal ascending aorta are promising [7,8], further clinical evidence is needed regarding the significantly more complex procedure of additional proximal aortic arch replacement. We here report a 15-year single-center experience of expanding the minimally invasive approach towards the proximal aortic arch compared to the well-established minimally invasive technique for isolated supracommissural ascending aorta replacement.

## 2. Patients and Methods

### 2.1. Patients

Between June 2009 and May 2023, 86 consecutive patients underwent elective minimally invasive ascending aorta replacement with or without concomitant proximal aortic arch replacement at our tertiary care center. Patient data including preoperative characteristics, intraoperative and postoperative course and complications as well as long-term postoperative survival were collected prospectively in our institutional database and analyzed retrospectively for this study. Postoperative follow-up was performed during regular visits in our outpatient clinic. Patients requiring concomitant aortic root or aortic valve surgery were excluded from the study. The necessity for concomitant procedures of the atrioventricular valves or coronary bypass grafting as well as emergency situations and redo operations contraindicate the upper hemisternotomy approach in our institution.

### 2.2. Study Design and Variables

For a retrospective single-center analysis, the study population was divided into two groups. The first group included all patients who received isolated minimally invasive ascending aorta replacement, while the second group consisted of patients in which additional proximal aortic arch repair was performed. For sorting into the groups, the following definitions were used: isolated ascending aorta replacement was defined as implantation of a straight aortic graft with a proximal suture line positioned on or above the sinotubular junction and a distal suture line located proximal to the brachiocephalic trunk within the Ishimaru zone 0, facilitating continuous cross-clamping of the distal ascending aorta and continuous body perfusion via direct aortic cannulation. In contrast, the distal suture line of proximal aortic arch replacement was placed more distally between zone 0 in the outer curvature and zone 1 in the inner curvature of the aortic arch to facilitate narrowing of the proximal arch while avoiding reimplantation of the brachiocephalic trunk. In these cases of hemiarch replacement, an open distal anastomosis is necessary, requiring hypothermic circulatory arrest.

For this study, preexisting coronary artery disease was defined as any treated or untreated flow-limiting coronary artery obstruction diagnosed by coronary angiography. Chronic kidney disease was defined as a decreased glomerular filtration rate of less than 60 mL/min (1.73 m^2^) for at least 3 months [18]. Any prior surgical procedures involving the heart or large intrathoracic vessels were summarized as cardiac preoperations. Chronic type A aortic dissection was defined as any aortic dissection involving the ascending aorta persisting for more than 14 days after the initial intima rupture [19].

### 2.3. Surgical Techniques

For both isolated ascending aorta replacement and hemiarch replacement, an upper J-shaped hemisternotomy to the third or fourth intercostal space was used. After applying the sternal retractor, pericardial stay sutures were placed to enhance exposure, and heparinization with a dose of 400–500 IU/kgBWT (body weight) was initiated. After reaching an activated clotting time of >400 s, distal ascending aortic and direct right atrial cannulation were performed in standard fashion. Venting was established through the main pulmonary artery in this minimally invasive setting. Continuous carbon dioxide insufflation was initiated, extracorporeal circulation was started, the aorta was cross-clamped, and cold blood cardioplegia was administered. For hemiarch replacement, cooling was initiated with a target bladder temperature of 25 °C. The aorta was opened and resected proximally until directly above the sinotubular junction. For isolated supracommissural ascending aorta replacement, Terumo Aortic Gelweave^®^ (Terumo Aortic, Inchinnan, UK) straight aortic grafts were used, and distal and proximal anastomoses were made using running Prolene^®^ sutures (Prolene, Somerville, NJ, USA). For additional hemiarch replacement, the Terumo Aortic Gelweave Anteflo^®^ aortic grafts were used. The decision for or against selective cerebral perfusion was based on the expected circulatory arrest time taking into consideration anatomical features including accessibility of the distal anastomosis site and quality of the aortic tissue. If necessary, antegrade cerebral perfusion via direct cannulation of the brachiocephalic trunk (*n* = 15) or retrograde cerebral perfusion via the superior vena cava (*n* = 5) was performed. For expected circulatory arrest times of less than 10 min, no selective cerebral perfusion was used (*n* = 20). The distal anastomosis for hemiarch replacement was performed in an oblique fashion reaching from zone 0 in the outer curvature to zone 1 in the inner curvature. After this, the arterial line of the extracorporeal circulation was placed in the side branch of the Anteflo^®^ prosthesis (Terumo Aortic, Inchinnan, UK), and extracorporeal circulation was continued. Subsequently, the proximal prosthesio-aortic anastomosis at the level of the sinotubular junction was performed. After careful deairing, the patients were weaned from extracorporeal circulation in standard fashion in both groups.

### 2.4. Statistical Analysis

IBM SPSS Statistics 28 (IBM Corp., Armonk, NY, USA, 1989, 2021) was used for statistical analyses. The Kolmogorov–Smirnov test was used to test for normal distribution, and normally distributed data are given as mean and standard deviation (SD). Median and interquartile range (Q1–Q3) are used to present non-normally distributed data. Homoscedasticity was tested using the Lavene test. The T-test or the Mann–Whitney test were used to compare continuous variables. Categorial variables are shown as total numbers (*n*) and percentages. The Kaplan–Meier survival estimates including the log-rank test were used to analyze survival. Differences were considered significant at *p* < 0.05.

## 3. Results

Proximal aortic arch replacement was performed in 40 (46.5%) of 86 patients included in this study. With a median patient age of 69 years (IQR 64.4–75.4 years) vs. 64.5 (IQR 53.5–74.4, *p* = 0.029), patients in the hemiarch group were slightly older compared to the isolated ascending aorta replacement group. Likewise, they more often had a positive history of preexisting coronary artery disease, while bicuspid aortic valve morphology was distinctively more frequent in patients undergoing isolated ascending aorta replacement (23.5% vs. 7.5%, *p* = 0.04) (Table 1). While the indication for surgery in the vast majority of patients in both groups was aortic aneurysm, each case of penetrating aortic ulcer and chronic type A aortic dissection was present in the group that underwent proximal aortic arch replacement.

Operation time and bypass time were significantly longer in the hemiarch group. Here, the median hypothermic circulatory arrest time was 9.5 min (IQR 7.0–18.0 min). In one case (2–5%) in the hemiarch group, conversion to full sternotomy was necessary due to bleeding that was not controllable via the minimally invasive access point (Table 2).

In the early postoperative course, ventilation time after proximal aortic arch replacement was approximately twice as long as after isolated ascending aorta replacement (20.4 ± 7.0 vs. 10.3 ± 4.8 min, *p* = 0.009). Compared to that, the length of stay at the intensive care unit and postoperative hospital stay differed less distinctively and did not reach statistical significance (Table 3). Within the study population, no cases of postoperative dialysis requirement or paraplegia and paraparesis were found in both groups.

**Figure 1:**
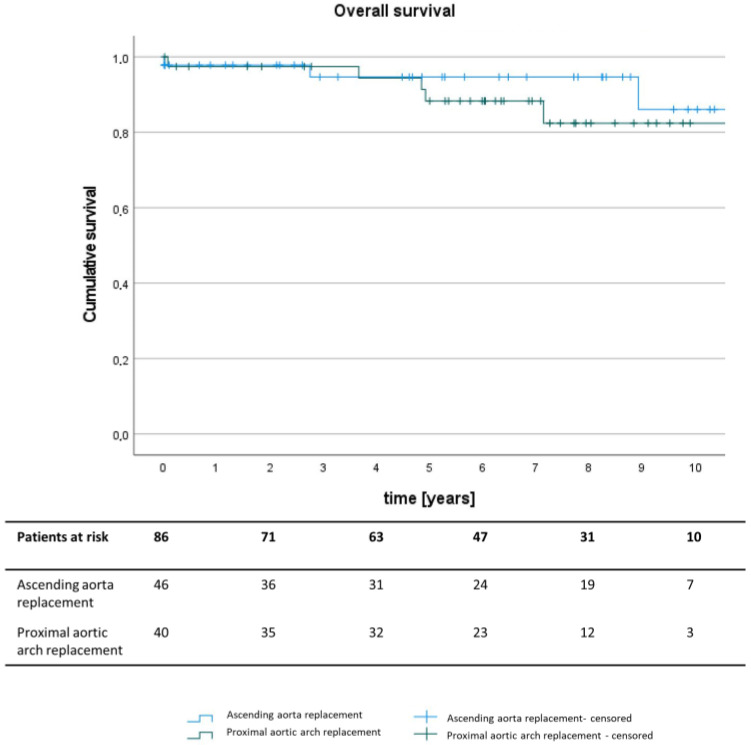
Kaplan–Meier analysis of long-term overall postoperative survival after isolated ascending aorta replacement (blue) or concomitant hemiarch replacement (green). Censored data are marked by horizontal lines.

The survival analysis revealed one in-hospital death (2.2%) in the isolated ascending aorta replacement group as well as one death after discharge within the first postoperative month (2.5%) in the hemiarch group (*p* = 0.920 for 30-day mortality). Median follow-up time for long-term survival was 6.0 years. In the Kaplan–Meier analysis, no significant differences between the two groups were found with an overall survival of over 80% after 10 years of follow-up in both groups (log rank = 0.441) (Figure 1).

## 4. Discussion

The main findings of this study can be summarized as follows: (1) Both isolated ascending aorta replacement and hemiarch replacement could be performed safely through a minimally invasive access site with a low conversion rate to full sternotomy. (2) Expanding the minimally invasive approach towards proximal aortic arch replacement prolonged the ventilation time but did not significantly increase the duration of the ICU or hospital stay. (3) In long-term follow-up, overall survival did not differ significantly between the two groups.

The safety and feasibility of minimally invasive ascending aorta replacement either alone or in combination with aortic valve or aortic root replacement has been demonstrated in different studies over the last few years [8,9,12,14]. In a propensity score-matched analysis of patients undergoing aortic valve replacement with supracommissural ascending aorta replacement via a minimally invasive partial sternotomy compared to classical full sternotomy, Haunschild et al. found similar results concerning safety and long-term durability outcomes [8]. They reported an in-hospital mortality of 1.7% in the minimal access group, which is within the range of our findings of an in-hospital mortality rate of 2.2% for the isolated ascending aorta replacement group and 1.2% for the entire study population. With these values, the early mortality after ascending aorta replacement in this study is similar to previously published mortality rates for minimally invasive approaches and within the range of the early mortality reported for ascending aorta replacement through full sternotomy [8,20].

However, the above-mentioned studies were mainly focused on the replacement of the proximal ascending aorta and did not involve hemiarch repair. Since replacement of the proximal aortic arch requires an open distal anastomosis, hypothermic circulatory arrest and not infrequently selective cerebral perfusion, first approaches for minimally invasive aortic surgery were limited to the proximal ascending aorta. In this field, a relatively broad basis of clinical evidence has been provided to date [3]. In contrast, chronologically later expansion of the minimally invasive technique towards the proximal has mainly been reported in the form of subgroup analyses with smaller case numbers among heterogeneous study populations to date: here, Kaneko et al. reported a 9-year single center experience of minimally invasive aortic valve and ascending aortic surgery [11]. Within their study cohort of 109 patients, 8 patients underwent concomitant proximal aortic arch replacement. In a combined subgroup analysis together with other concomitant procedures including Bentall or David operations, they reported an early mortality of 3.7% with no cases of conversion to full sternotomy. Although no specific subgroup analysis for proximal aortic arch replacement was performed by Kaneko et al. due to the limited number of cases, these results are consistent with our findings regarding postoperative complication rates. However, we saw one conversion to full sternotomy in our hemiarch study population. For long-term survival, they report a survival rate of approximately 80% after 10 years for their entire study population, which is comparable to the overall survival rate for both groups in the analysis presented here.

In another report by Deschka et al., 11 out of 50 patients undergoing minimally invasive surgery of the aortic root and ascending aorta received concomitant proximal aortic arch replacement [16]. While they report excellent short-term outcomes, they too report comparatively long postoperative ventilation periods with a mean ventilation time of 29 h. This observation is consistent with our findings of comparatively long postoperative ventilation times after proximal aortic arch replacement and may be due to prolonged warming periods after hypothermic circulatory arrest.

In a more recent propensity score-matched analysis including a total of 36 patients, Wu et al. compared minimally invasive techniques to classical full sternotomy for complex procedures in the setting of aortic dissection [21]. In addition to hemiarch replacement, their cohort also included 13 cases of total aortic arch replacement via upper hemisternotomy, which were performed using the arch-first technique, involving reconstruction of the supraaortic vessels prior to frozen elephant trunk implantation.

In this combined study population of different, more complex concomitant procedures, they found similar postoperative complication rates compared to isolated ascending aorta replacement or classical full sternotomy approaches; in-hospital mortality was overall low with one death recorded in the hemisternotomy group. Here, cross-clamp times were slightly longer in the hemisternotomy group. However, in their experience, the length of ICU stay and hospital stay were even shorter when using the minimally invasive approach. Our findings regarding the postoperative complication rates after hemiarch replacement confirm these reports. Compared to isolated ascending aorta replacement, performing proximal aortic arch replacement did not result in higher rates of postoperative complications or prolonged length of stay in our analysis.

## 5. Limitations and Conclusions

As this study is a retrospective single-center analysis, certain limitations need to be considered including a possible selection bias. Additionally, urgent and emergent cases were excluded from the analysis, and data obtained from the elective setting cannot be extrapolated to more urgent procedures. Additionally, minimally invasive proximal aortic arch replacement was not compared to hemiarch replacement via classical full sternotomy in this study. Lastly, minor differences between the study groups may not have been recorded or reached statistical significance due to the limited number of cases included in the study, which furthermore prohibited subgroup analyses of different age groups within the study population.

Expanding the indication for elective minimally invasive aortic surgery towards the proximal aortic arch resulted in comparable postoperative complication rates, length of hospital stay and overall long-term survival compared to the well-established minimally invasive isolated supracommissural ascending aorta replacement. This evidence suggests that even the more complex operation of hemiarch replacement requiring an open distal anastomosis and circulatory arrest can be performed safely through a minimally invasive access point with a similar outcome compared to isolated ascending aorta replacement.

## Figures and Tables

**Table 1:** Preoperative characteristics. COPD = chronic obstructive pulmonary disease. Values in bold mark statistically significant differences with *p* < 0.05.

Characteristics	Ascending Aorta Replacement (*n* = 46)	Proximal Aortic Arch Replacement (*n* = 40)	*p*-Value
Sex (male)	22 (47.8%)	14 (35.0%)	0.229
Age (years)	64.5 (53.5–74.4)	69.0 (64.4–75.4)	**0.029**
Coronary artery disease	6 (13.0%)	14 (35.0%)	**0.016**
Atrial fibrillation	5 (10.9%)	4 (10.0%)	0.895
Pulmonary artery embolism	3 (6.5%)	1 (2.5%)	0.337
Persistent foramen ovale	2 (4.3%)	0 (0.0%)	0.182
Preoperative stroke	1 (2.2%)	3 (7.5%)	0.242
Diabetes	2 (4.3%)	2 (5.0%)	0.886
Chonic kidney disease	1 (2.2%)	3 (7.5%)	0.242
COPD	4 (8.7%)	4 (10.0%)	0.835
Bicuspid aortic valve	11 (23.5%)	3 (7.5%)	**0.040**
Aortitis	2 (4.3%)	1 (2.5%)	0.641
Chronic aortic dissection	0 (0.0%)	1 (2.5%)	0.281
Penetrating aortic ulcer	0 (0.0%)	1 (2.5%)	0.281
Cardiac preoperation	0 (0.0%)	1 (2.5%)	0.281

**Table 2:** Intraoperative characteristics. Values in bold mark statistically significant differences with *p* < 0.05.

Characteristics	Ascending Aorta Replacement (*n* = 46)	Proximal Aortic Arch Replacement (*n* = 40)	*p*-Value
Concomitant cardiac procedure	3 (6.5%)	2 (5.0%)	0.764
Graft size (mm)	28 (26–30)	30 (28–30)	0.130
Operation time (min)	168 (143–204)	223 (207–243)	**<0.001**
Bypass time (min)	77 (65–102)	123 (104–139)	**<0.001**
Cross-clamp time (min)	47 (39–57)	51 (40–67)	0.301
Conversion to full sternotomy	0 (0.0%)	1 (2.5%)	0.281

**Table 3:** Postoperative characteristics. ICU = intensive care unit. Values in bold mark statistically significant differences with *p* < 0.05.

Characteristics	Ascending Aorta Replacement (*n* = 46)	Proximal Aortic Arch Replacement (*n* = 40)	*p*-Value
Ventilation time (hours)	10.3 ± 4.8	20.4 ± 7.0	**0.009**
ICU stay (days)	2.02 ± 2.1	2.83 ± 2.8	0.134
Hospital stay (days)	8 (7–11)	10 (8–13)	0.111
Reanimation (ventricular fibrillation)	1 (2.2%)	2 (5.0%)	0.476
Delirium	0 (0.0%)	2 (5.0%)	0.125
Atrial fibrillation	6 (13.0%)	5 (12.5%)	0.940
Re-thoracotomy	0 (0.0%)	2 (5.0%)	0.125
Tracheotomy	0 (0.0%)	1 (2.5%)	0.281
Stroke	1 (2.2%)	3 (7.5%)	0.242

## Data Availability

Data used in this study are available from the corresponding author upon request.
